# Multivariate analysis of eshnan (*Seidlitzia rosmarinus* Boiss.) based on morphological characterizations

**DOI:** 10.1002/fsn3.2964

**Published:** 2022-07-14

**Authors:** Farhad Mirheidari, Ali Khadivi

**Affiliations:** ^1^ Department of Horticultural Sciences, Faculty of Agriculture and Natural Resources Arak University Arak Iran

**Keywords:** breeding, conservation, germplasm, leaf size, *Seidlitzia rosmarinus*

## Abstract

*Seidlitzia rosmarinus* Boiss. has been identified as one of the potential species that could be used for rehabilitating degraded desert rangelands and salt‐affected soils due to its high salinity resistance and soil‐stabilizing ability. Morphological variation of 144 accessions of this species from 14 regions of the Isfahan province, Iran was investigated. The accessions studied were significantly different in terms of the traits recorded. Three forms of plant growth habit were observed, including spreading bush, erect bush, and shrub. The range of leaf dimensions was as follows: terminal leaf length: 1.57–7.22 mm, terminal leaf width: 0.91–3.34 mm, basal leaf length: 11.84–45.27 mm, and basal leaf width: 1.32–4.18 mm. Fruit diameter (with wings) ranged from 0.19 to 12.91 mm, and 100‐fruits dry weight varied between 0.11 and 0.76 g. A dendrogram created using Euclidean distances and the Ward's method revealed two main clusters. The obtained data revealed the morphological diversity within the studied populations. The reason for such a high diversity can be explained by a low probability of gene flow among the studied accessions. This is the first report on the application of morphological characteristics in the evaluation of the phenotypic variation of *S. rosmarinus*. This study presented a high phenotypic diversity of *S. rosmarinus* germplasm that could provide useful information for conservation and selection of cross‐parents in breeding.

## Graphical abstract

This study presented the high phenotypic diversity of *Seidlitzia rosmarinus* Boiss. germplasm that could provide useful information for conservation and selection of cross‐parents in breeding.

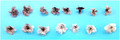

## 1 INTRODUCTION

Eshnan (*Seidlitzia rosmarinus* Boiss., Synonym: *Salsola rosmarinus* [Bunge ex Boiss.] Eig) in the Amaranthaceae, is a perennial halophytic shrub that grows up to 80 cm in height and inhabits sandy plains, sabkha (salt flats), wadi (riverine gulches), and drainage channels that have alkaline and saline soils (Jongbloed, 2003). It is widely distributed in Iran, Jordan, the United Arabic Emirates, Saudi Arabia, Kuwait, Qatar, Bahrain, Jordan, Afghanistan, and Central Asia (Deymeh et al., 2012; Hadi, 2009; Jongbloed, 2003; Sagheb‐Talebi et al., 2014). The *S. rosmarinus* has been identified as one of the species with high potential that could be used for rehabilitating degraded desert rangelands and salt‐affected soils due to its high salinity resistance and soil‐stabilizing ability (Amiraslani & Dragovich, 2011; Jafari et al., 2003; Mahmoodi et al., 2013) besides being frequently grazed by camels (Koocheki & Mahalati, 1994).

The *S. rosmarinus* has been used as forage for a long time (Koocheki & Mahalati, 1994). Although the nutritional values of halophytes such as *S. rosmarinus* are relatively good, they make palatable forage when mixed with other pasture plants (Swingle et al., 1996). In ancient times, Iranian people used to make holy bonfire using plants growing in saline soils. They used *S. rosmarinus* ashes as detergent to wash their bodies and their clothing. When mixed with oil or suet, it makes high‐quality soap. Today, the ash of this plant is a source of alkaline materials, used in soap and detergent industries. The ash has also antiseptic and antibacterial properties. Root tissues of *S. rosmarinus* have a high capacity to absorb large amounts of soil alkali metals such as Na+ and K+, which are subsequently transferred to the shoots. It seems the main mechanism of salt resistance in this plant is tolerance. Large amount of sodium is accumulated in cell vacuoles. The ash contains a large amount of sodium and potassium carbonates (Koocheki & Mahalati, 1994).

Phenotypic diversity in plants is required for populations to evolve in response to environmental changes, and its maintenance is crucial for long‐term species survival. Therefore, knowledge of phenotypic variation of an endangered species under different environments is the prerequisite for understanding its genetic variation pattern, fitness, and evolutionary capacity to adapt to environmental changes, and it is crucial for their in situ conservation and management (Yang et al., 2013). Morphological evaluation and characterization are the first steps for the description and classification of germplasm (Badenes et al., 2000).

Until now, there is no published report on the evaluation of genetic diversity of *S. rosmarinus*. The main objective of the present work was the evaluation of morphological diversity of the *S. rosmarinus* populations in the Isfahan province, Iran to select the individuals to be used in the breeding programs.

## 2 MATERIALS AND METHODS

### 2.1 Plant material

Morphological variation of 144 accessions of *S. rosmarinus* from 14 regions of the Isfahan province, Iran was investigated. Geographical coordinates and altitude corresponding to collection sites are shown in Table 1. The appropriate distances were considered between the accessions in each collection site to avoid the possibility of sampling and collecting clones of the selected plants.

**TABLE 1:** Geographical description for collection sites of *S. rosmarinus* accessions studied from Isfahan province, Iran

No.	Area	Latitude (N)	Longitude (E)	Altitude (m)	Sample size
1	Khoya	32°33′00″	52°05′12″	1514	10
2	Shoor	32°39′34″	52°15′56″	1535	10
3	Zardenjan	32°34′38″	51°50′31″	1541	10
4	Nikabad	32°18′42″	52°10′57″	1559	10
5	Heidarabad	32°16′38″	52°17′42″	1548	10
6	Dastjerd	32°10′36″	52°37′25″	1476	10
7	Arisman	33°41′05″	51°49′72″	1094	10
8	Dehzire	33°44′55″	51°47′14″	1126	10
9	Badrood	33°40′36″	51°57′48″	1010	10
10	Ardestan	33°24′16″	52°22′47″	1132	10
11	Varzaneh	32°22′10″	52°32′22″	1468	10
12	Gavkhooni	32°24′41″	52°41′34″	1460	10
13	Harand	32°34′39″	52°26′38″	1563	10
14	Sagzi	32°40′45″	51°56′02″	1538	10

### 2.2 The characteristics evaluated

A total of 45 morphological and pomological traits (Table 2) were used for phenotypic evaluations. The traits such as dimensions of internode, leaf, fruit, and seed, were measured using a digital caliper. Fruit weight was measured using an electronic balance with 0.01 g precision. In addition, the remaining characteristics were qualitatively estimated based on rating and coding (Table 3).

**TABLE 2:** Statistical descriptive parameters for morphological traits used to study *S. rosmarinus* accessions

No.	Character	Unit	Min.	Max.	Mean	SD	CV (%)
1	Plant growth habit	Code	1	5	2.86	1.49	52.13
2	Plant growth vigor	Code	1	5	3.79	1.35	35.65
3	Plant height	cm	31	215	92.21	38.59	41.85
4	Canopy diameter	cm	45	420	174.57	66.62	38.16
5	Canopy density	Code	1	5	3.66	1.41	38.47
6	Branching	Code	1	5	3.90	1.23	31.56
7	Branch density	Code	1	5	3.84	1.23	31.90
8	Branch flexibility	Code	1	5	2.50	1.38	55.24
9	Main shoot color	Code	1	7	6.00	1.24	20.58
10	Main shoot diameter	mm	10.32	320.48	86.16	53.21	61.75
11	Upper lateral shoot diameter	mm	5.22	155.26	36.22	25.26	69.75
12	Lower lateral shoot diameter	mm	1.05	22.82	12.57	3.18	25.30
13	Current year shoot color	Code	1	5	2.09	1.03	49.19
14	Perennial shoot color	Code	1	5	3.34	1.27	37.96
15	Main shoot initial internode length	mm	11.35	310.10	70.08	57.21	81.63
16	Main shoot medial internode length	mm	14.14	750.30	82.34	116.64	141.66
17	Main shoot terminal internode length	mm	0.10	6.74	2.22	1.42	64.14
18	Lateral shoot initial internode length	mm	9.82	49.34	21.77	7.21	33.11
19	Lateral shoot medial internode length	mm	10.88	70.33	29.21	9.67	33.10
20	Lateral shoot terminal internode length	mm	0.13	5.10	1.59	1.10	69.43
21	Leaf density	Code	1	5	3.56	1.32	36.94
22	Leaf color	Code	1	5	3.11	1.29	41.38
23	Terminal leaves shape	Code	1	5	1.46	1.21	82.74
24	Terminal leaf length	mm	1.57	7.22	3.73	1.13	30.38
25	Terminal leaf width	mm	0.91	3.34	1.81	0.44	24.25
26	Basal leaves shape	Code	1	3	1.46	0.84	57.74
27	Basal leaf length	mm	11.84	45.27	27.35	6.38	23.33
28	Basal leaf width	mm	1.32	4.18	2.55	0.57	22.20
29	Flowering date	Date	Late June	Mid‐Aug	4.57	3.24	70.79
30	Flower density	Code	1	5	3.29	1.39	42.25
31	Flower stamen color	Code	1	5	3.41	1.16	34.11
32	Hypanthium diameter	mm	0.57	2.76	1.57	0.35	22.48
33	Flower number in leaf axil	Number	1	3.80	2.91	0.58	19.76
34	Fruit ripening date	Date	Mid‐Oct	Early Nov	2.71	1.67	61.70
35	Fruit diameter (with wings)	mm	0.19	12.91	8.54	2.07	24.25
36	100‐fruits dry weight	g	0.11	0.76	0.38	0.10	27.11
37	Seed wings number	Number	4	7	5.06	0.48	9.41
38	Seed wing shape	Code	1	3	2.43	0.91	37.33
39	Seed wing apex	Code	1	3	1.60	0.92	57.50
40	Seed wing length	mm	0.07	8.87	3.84	1.62	42.16
41	Seed wing width	mm	0.30	7.28	4.34	1.55	35.78
42	Seed wing color	Code	1	5	3.54	1.29	36.38
43	Seed color	Code	1	3	1.71	0.96	56.26
44	Seed diameter	mm	0.54	4.32	2.13	0.48	22.54
45	Seed thickness	mm	0.36	2.96	1.13	0.56	49.12

**TABLE 3:** Frequency distribution for the measured qualitative morphological characteristics in the studied *S. rosmarinus* accessions

Character	Frequency (no. of accessions)					
	1	3	5	7	9	11
Plant growth habit	Spreading bush (44)	Erect bush(62)	Shrub form (34)	–	–	–
Plant growth vigor	Low (15)	Moderate (55)	High (70)	–	–	–
Canopy density	Low (19)	Moderate (56)	High (70)	–	–	–
Branching	Low (9)	Moderate (59)	High (72)	–	–	–
Branch density	Low (9)	Moderate (63)	High (68)	–	–	–
Branch flexibility	Low (55)	Moderate (65)	High (20)	–	–	–
Main shoot color	Light green (1)	Cream (6)	Light brown (55)	Brown (78)	–	–
Current year shoot color	Light green (65)	Cream (74)	Light brown (1)	–	–	–
Perennial shoot color	Cream (18)	Light brown (80)	Brown (42)	–	–	–
Leaf density	Low (16)	Moderate (69)	High (55)	–	–	–
Leaf color	Light green (25)	Green (82)	Dark green (33)	–	–	–
Terminal leaves shape	Short wand (121)	Long wand (6)	Cylindrical (13)	–	–	–
Basal leaves shape	Long wand (108)	Cylindrical (32)	–	–	–	–
Flowering date	Late June (50)	Early July (10)	Mid‐July (30)	Late July (30)	Early August (10)	Mid‐August (10)
Flower density	Low (25)	Moderate (70)	High (45)	–	–	–
Flower stamen color	Light yellow (12)	Yellow (87)	Dark yellow (41)	–	–	–
Fruit ripening date	Mid‐October (60)	Late October (40)	Early November (40)	–	–	–
Seed wing shape	Oval (40)	Blowing (100)	–	–	–	–
Seed wing apex	Round (98)	Notched (42)	–	–	–	–
Seed wing color	White (15)	Pink (72)	Purple (53)	–	–	–
Seed color	Gray (90)	Black (50)	–	–	–	–

### 2.3 Statistical analysis

Analysis of variance (ANOVA) was performed to evaluate the variation among the accessions based on the traits measured using SAS software (SAS Institute, Cary, NC, USA, 1990). Principal component analysis (PCA) was used to investigate the relationship between the accessions and determine the main traits effective in genotype segregation using SPSS software (SPSS Inc., Chicago, IL, USA, Norusis, 1998). Hierarchical cluster analysis (HCA) was performed using Ward's method and Euclidean coefficient using PAST software (Hammer et al., 2001). The first and second principal components (PC1/PC2) were used to create a scatter plot with PAST software.

## 3 RESULTS AND DISCUSSION

The accessions studied were significantly different in terms of the traits recorded as revealed with ANOVA. Main shoot medial internode length had the highest CV (141.66%) followed by terminal leaves shape (82.74%) and main shoot initial internode length (81.63%). Out of 45 characteristics measured, only two of them showed the CV less than 20.00%, including flower number in leaf axil (19.76%), and seed wings number (9.41%) (Table 2). Thus, the CVs obtained confined the ANOVA results which showed significant differences among the accessions.

Three forms of plant growth habit were observed, including spreading bush (44 accessions), erect bush (62), and shrub (34). Plant growth vigor, canopy density, branching, and branch density were dominantly high (Table 3). Main shoot color was mostly brown (78 accessions), Current year shoot color was cream in most of the accessions (74), and perennial shoot color was light brown in most of the accessions (80) (Table 3). Plant height ranged from 31 to 215 cm, and canopy diameter varied from 45 to 420 cm. The main shoot diameter ranged from 10.32 to 320.48 mm, upper lateral shoot diameter varied from 5.22 to 155.26 mm, and lower lateral shoot diameter ranged from 1.05 to 22.82 mm (Table 2).

Terminal leaves shape was dominantly short wand (121 accessions), while basal leaves shape was long wand in the majority of accessions (108). The range of leaf dimensions was as follows: terminal leaf length: 1.57–7.22 mm, terminal leaf width: 0.91–3.34 mm, basal leaf length: 11.84–45.27 mm, and basal leaf width: 1.32–4.18 mm (Table 2).

**FIGURE 1:**
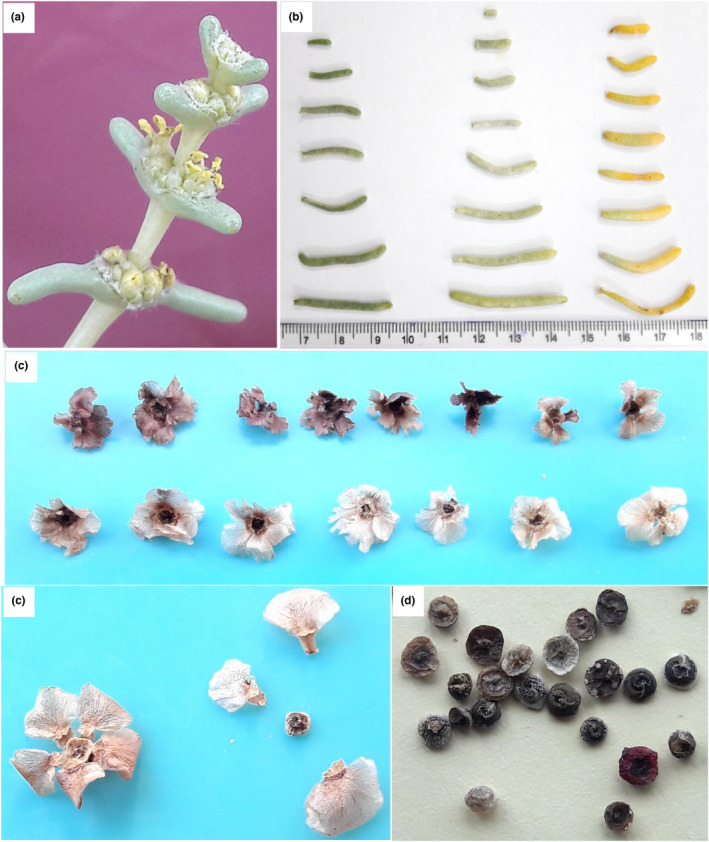
The pictures of different organs of *S. rosmarinus* accessions studied: (a) flower, (b) leaf, (c) fruit, and (d) seed.

The flowering date ranged from late June to Mid‐August. Flower density was low (25 accessions), moderate (70), and high (45). Flower stamen color was light yellow (12 accessions), yellow (87), and dark yellow (41). Fruits of 60 accessions were ripened in mid‐October, 40 accessions in late October, and 40 accessions in early November. Fruit diameter (with wings) ranged from 0.19 to 12.91 mm, and 100‐fruits dry weight varied between 0.11 and 0.76 g. Seed wing shape was predominantly blowing (100 accessions), seed wing apex was round the majority of accessions (98), and seed wing color of most of the accessions (72) was pink, and the range of seed wing number was 4–7. Also, the seed color was gray (90), and black (50) (Table 3). The range of seed‐related traits was as follows: seed wing length: 0.07–8.87 mm, seed wing width: 0.30–7.28 mm, seed diameter: 0.54–4.32 mm, and seed thickness: 0.36–2.96 mm (Table 2). The pictures of different organs of *S. rosmarinus* accessions studied are shown in Figure 1.

The most important variables influencing to distinguish the variations among the accessions were determined using the PCA. Eigenvalues >1.00 were highlighted as criteria to extract the main components, to determine the PC that showed the greatest value of diversity. The loaded values ≥0.50 were considered as significant for each factor, which showed 14 components with explaining 70.49% of the total variance (Table 4). The PC1 was positively correlated with plant growth habit (0.59), plant height (0.87), canopy diameter (0.82), main shoot diameter (0.85), upper lateral shoot diameter (0.83), and perennial shoot color (0.54), accounting for 10.22% of total variance. Five traits, including plant growth vigor (0.63), canopy density (0.82), branching (0.90), branch density (0.91), and branch flexibility (0.50), were significantly and positively correlated with PC2, accounting for 8.41% of total variance. Thus, PC1 and PC2 could be called as vegetative‐related traits. The PC3 was associated with fruit diameter (with wings) (0.85), seed wing length (0.90), seed wing width (0.89), and seed thickness (−0.68) accounting for 7.48% of total variance.

**TABLE 4:** Eigenvalues of the principal component axes from the PCA of the morphological characteristics in the studied *S. rosmarinus* accessions

Character	Component													
	1	2	3	4	5	6	7	8	9	10	11	12	13	14
Plant growth habit	0.59**	−0.08	0.07	−0.15	−0.06	0.05	0.03	−0.18	0.02	0.43	0.12	−0.26	0.06	−0.07
Plant growth vigor	0.44	0.63**	0.07	−0.11	0.11	0.09	−0.02	−0.04	0.08	0.12	0.15	0.08	−0.07	0.03
Plant height	0.87**	−0.02	0.13	0.06	0.11	−0.08	−0.04	−0.02	−0.05	0.03	−0.07	0.08	−0.03	0.01
Canopy diameter	0.82**	0.16	0.06	0.07	0.14	0.03	−0.12	0.05	−0.10	−0.11	−0.13	0.13	−0.02	0.00
Canopy density	0.09	0.82**	0.12	−0.01	−0.05	0.03	0.08	−0.06	0.04	−0.08	0.07	0.08	0.05	0.11
Branching	0.06	0.90**	0.06	0.03	−0.05	−0.07	0.06	0.00	−0.02	−0.06	−0.03	0.01	0.00	−0.04
Branch density	0.02	0.91**	0.09	0.02	0.02	−0.04	0.03	0.00	−0.05	−0.03	−0.07	0.04	0.02	−0.03
Branch flexibility	−0.20	0.50**	0.18	−0.26	−0.03	0.09	−0.08	0.07	0.38	0.00	−0.08	0.08	−0.01	−0.07
Main shoot color	0.49	0.26	0.09	0.07	0.10	0.23	−0.05	−0.03	−0.17	0.15	0.07	−0.36	−0.20	0.17
Main shoot diameter	0.85**	0.12	0.02	−0.04	−0.03	0.04	−0.07	0.05	0.19	−0.06	0.04	0.02	0.09	0.04
Upper lateral shoot diameter	0.83**	0.07	0.03	0.07	−0.11	0.03	0.01	0.02	0.20	−0.16	0.01	0.13	0.13	−0.04
Lower lateral shoot diameter	−0.22	0.17	−0.10	0.18	−0.11	0.56**	0.11	0.11	0.16	−0.20	0.17	−0.03	−0.26	0.10
Current year shoot color	0.04	−0.17	−0.11	0.17	−0.21	−0.25	−0.45	−0.10	−0.20	−0.01	0.45	−0.30	0.19	−0.02
Perennial shoot color	0.54**	0.01	0.25	0.11	0.23	0.04	0.00	0.00	−0.16	0.10	−0.11	−0.27	−0.25	0.18
Main shoot initial internode length	0.44	−0.13	0.05	0.09	−0.10	−0.20	0.06	−0.01	0.62**	0.03	0.05	0.18	−0.21	0.03
Main shoot medial internode length	0.03	0.12	−0.03	0.02	0.07	−0.02	0.01	−0.15	0.74**	0.11	−0.20	−0.14	−0.03	−0.03
Main shoot terminal internode length	−0.10	−0.12	0.08	0.15	0.71**	0.20	−0.12	−0.01	−0.12	−0.11	0.12	−0.02	0.25	−0.07
Lateral shoot initial internode length	0.11	−0.12	−0.09	0.11	−0.04	0.69**	0.03	0.04	−0.09	−0.10	−0.06	0.00	0.05	−0.08
Lateral shoot medial internode length	0.05	0.10	0.19	−0.11	0.12	0.64**	−0.22	−0.29	−0.07	0.16	−0.11	0.01	0.05	0.05
Lateral shoot terminal internode length	−0.08	0.14	−0.20	−0.07	0.43	−0.01	0.37	0.22	0.07	0.42	−0.01	−0.11	0.29	0.00
Leaf density	0.12	0.49	0.06	0.27	0.13	−0.08	0.02	0.25	0.07	0.10	0.12	0.35	−0.32	−0.08
Leaf color	0.17	0.31	−0.08	0.03	0.02	0.00	−0.06	0.02	−0.07	0.00	0.08	0.62**	0.08	−0.09
Terminal leaves shape	−0.25	0.13	−0.11	−0.09	0.02	−0.01	−0.04	−0.30	0.15	−0.36	−0.30	−0.21	0.12	−0.15
Terminal leaf length	−0.08	−0.03	−0.18	0.02	0.06	−0.20	−0.07	0.75**	0.00	−0.02	0.05	−0.17	0.22	0.13
Terminal leaf width	0.05	0.00	0.08	0.02	−0.20	0.10	0.05	0.79**	−0.15	0.15	−0.10	0.16	0.02	−0.03
Basal leaves shape	0.06	−0.07	−0.13	−0.10	−0.07	0.05	−0.08	0.05	0.14	−0.07	−0.81**	−0.09	0.07	0.03
Basal leaf length	0.10	−0.13	−0.11	0.20	0.35	0.45	−0.27	0.01	−0.05	0.16	0.07	0.15	0.27	−0.12
Basal leaf width	−0.12	−0.02	0.01	−0.11	0.15	0.34	−0.37	0.20	−0.16	−0.02	0.46	0.31	0.12	0.15
Flowering date	0.02	−0.08	0.16	0.49	0.43	0.33	−0.32	−0.15	−0.33	−0.18	0.03	0.11	−0.01	−0.03
Flower density	−0.01	0.17	−0.09	0.59**	−0.08	0.07	0.21	0.06	0.04	0.40	0.14	0.16	−0.04	0.20
Flower stamen color	−0.12	−0.09	0.13	0.26	−0.20	−0.12	0.27	−0.29	0.25	0.17	0.00	−0.02	0.16	−0.34
Hypanthium diameter	0.14	0.11	−0.19	0.57**	−0.05	0.18	−0.01	0.31	0.13	−0.13	0.13	−0.16	0.14	−0.03
Flower number in leaf axil	0.03	0.14	−0.18	0.29	−0.18	0.21	0.06	−0.13	−0.08	0.10	0.08	0.52**	0.19	0.39
Fruit ripening date	0.03	−0.17	0.16	0.84**	0.19	0.02	0.02	−0.07	−0.03	0.00	−0.05	0.08	0.05	−0.06
Fruit diameter (with wings)	0.02	0.14	0.85**	0.02	0.03	−0.02	0.16	−0.05	0.05	−0.04	0.12	−0.06	−0.08	−0.02
100‐fruits dry weight	−0.04	0.07	−0.16	−0.19	0.06	−0.01	0.46	−0.03	0.28	−0.22	0.44	−0.08	0.21	0.15
Seed wings number	−0.05	−0.03	−0.07	−0.10	−0.04	−0.03	−0.02	−0.16	0.09	−0.07	0.00	−0.12	−0.73**	−0.06
Seed wing shape	−0.22	0.01	−0.08	0.00	−0.59**	0.05	−0.18	0.11	−0.26	−0.18	0.13	0.17	0.12	−0.13
Seed wing apex	−0.25	−0.17	−0.07	−0.02	−0.55**	0.24	0.08	0.14	0.02	0.12	−0.12	−0.15	0.23	−0.10
Seed wing length	0.14	0.08	0.90**	−0.10	0.05	−0.01	−0.01	0.02	−0.05	0.05	−0.04	−0.02	−0.02	0.01
Seed wing width	0.10	0.17	0.89**	0.08	−0.01	0.00	0.02	−0.04	−0.13	−0.01	−0.03	0.02	0.06	0.01
Seed wing color	−0.12	−0.07	0.14	0.03	−0.02	−0.07	−0.05	0.06	0.11	0.74**	−0.02	0.02	0.06	−0.06
Seed color	0.05	−0.04	0.05	0.00	0.04	−0.07	−0.03	0.07	0.01	−0.03	0.01	−0.04	0.05	0.88**
Seed diameter	−0.13	0.05	0.06	0.12	−0.06	−0.09	0.84**	−0.02	−0.07	0.01	0.04	0.00	−0.01	−0.06
Seed thickness	−0.13	0.00	−0.68**	−0.05	−0.06	0.03	0.19	0.04	−0.23	−0.26	−0.07	0.09	−0.12	−0.04
Total	4.60	3.78	3.36	2.26	2.09	2.09	1.92	1.91	1.87	1.71	1.67	1.63	1.46	1.36
% of Variance	10.22	8.41	7.48	5.02	4.65	4.64	4.27	4.25	4.15	3.81	3.71	3.63	3.25	3.01
Cumulative %	10.22	18.63	26.10	31.13	35.77	40.41	44.68	48.94	53.09	56.89	60.61	64.23	67.48	70.49

**Eigenvalues ≥0.50 are significant at the *p* ≤ .01 level.

**FIGURE 2:**
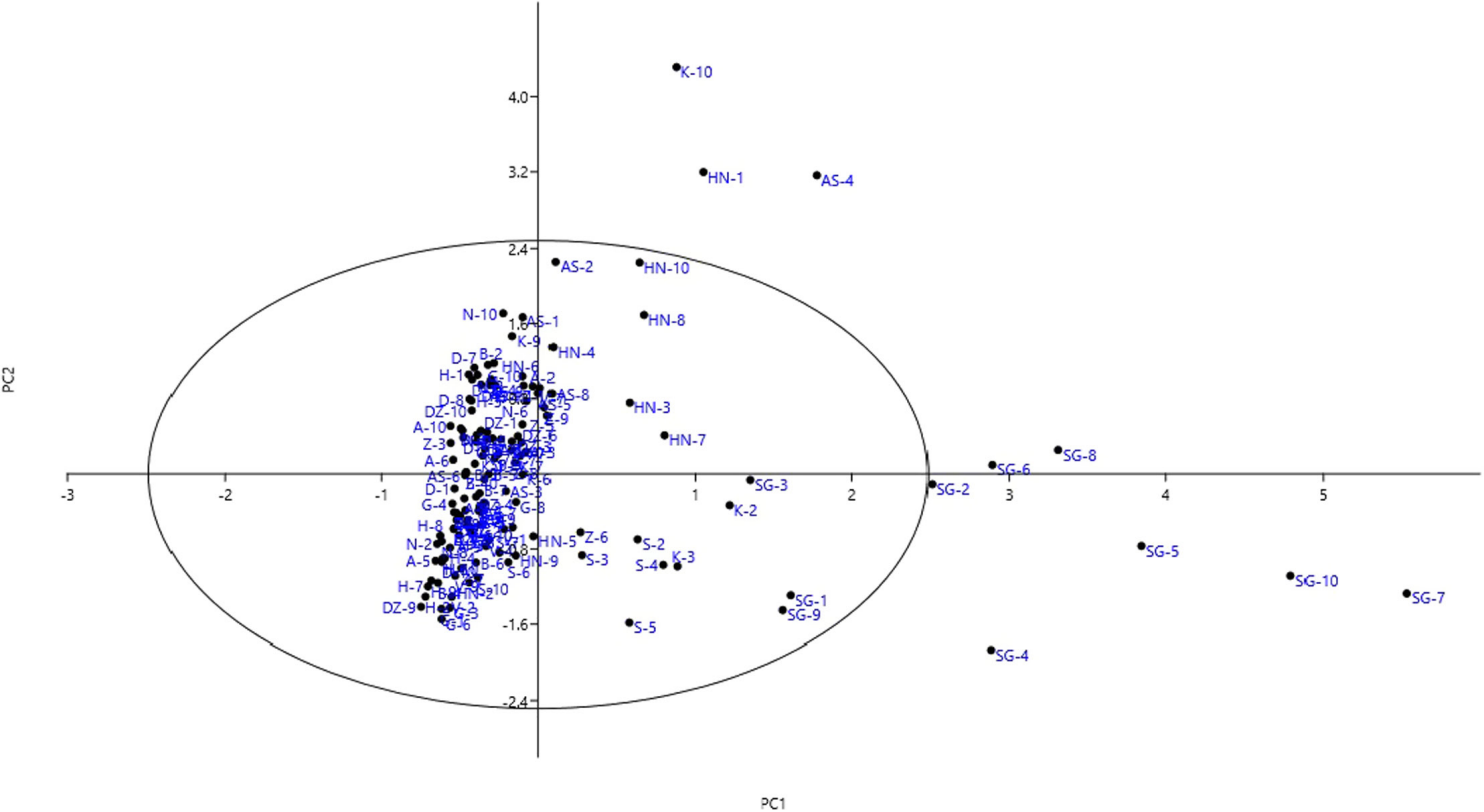
Scatter plot for the studied *S. rosmarinus* accessions based on PC1/PC2. The symbols represent the accessions of each area in the plot, including khoya (K), Shoor (s), Zardenjan (Z), Nikabad (n), Heidarabad (h), Dastjerd (D), Arisman (a), Dehzire (DZ), Badrood (b), Ardestan (AS), Varzaneh (v), Gavkhooni (g), Harand (HN), and Sagzi (SG).

**FIGURE 3:**
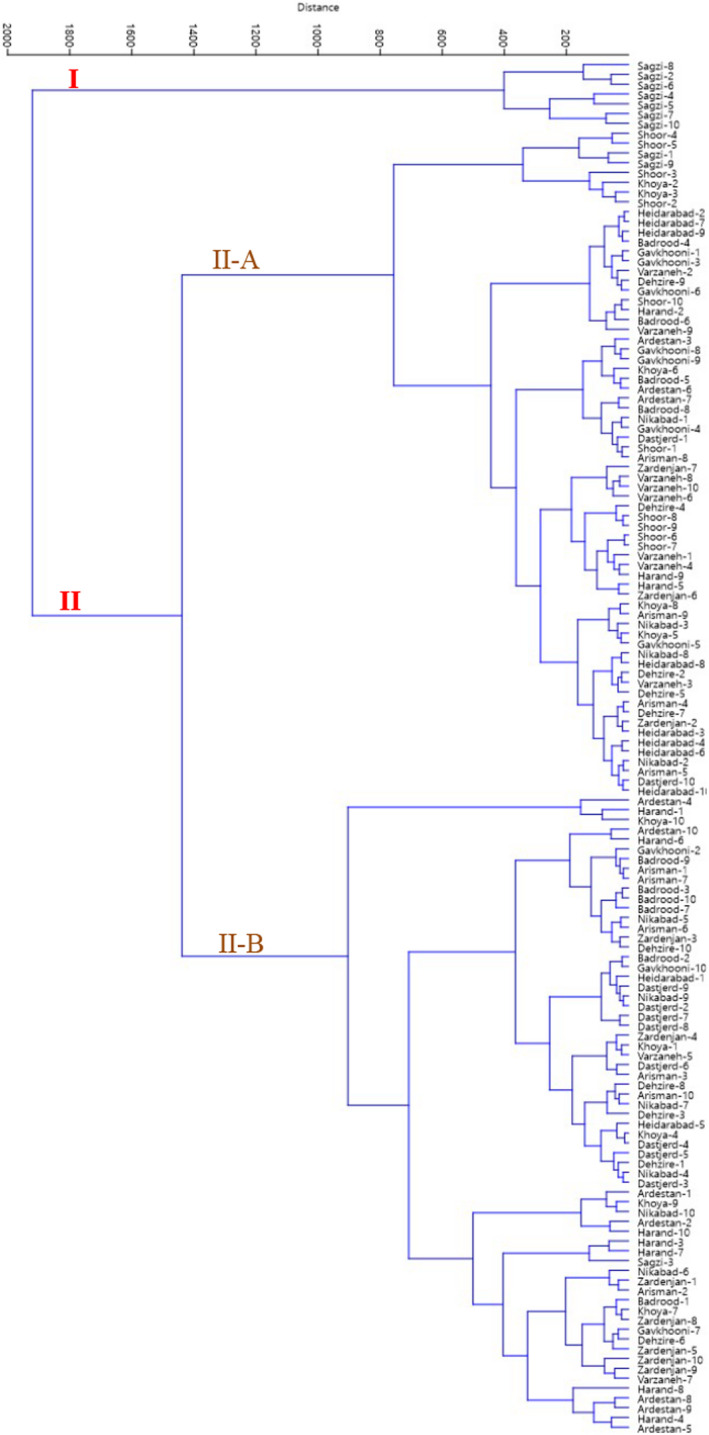
Ward cluster analysis of the studied *S. rosmarinus* accessions based on morphological traits using Euclidean distances.

**FIGURE 4:**
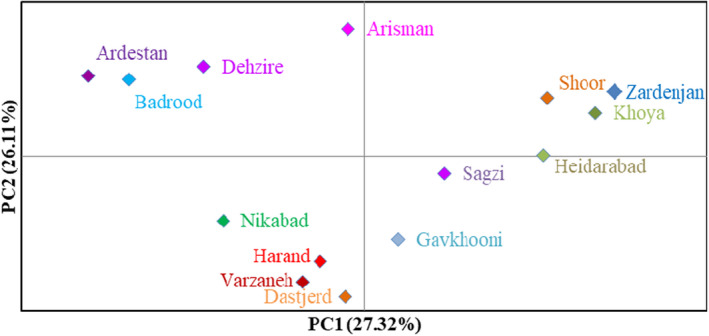
Bi‐plot for the studied populations of *S. rosmarinus* based on the morphological characteristics.

The projection of the studied accessions based on the PC1/PC2 plot reflected the relationship among them in terms of phenotypic resemblance (Figure 2). By starting from negative toward positive values of PC1, the accessions showed gradual increases in plant growth habit, plant height, canopy diameter, main shoot diameter, upper lateral shoot diameter, and perennial shoot color. Furthermore, starting from negative to positive values of PC2, the accessions indicated gradual increases in plant growth vigor, canopy density, branching, branch density, and branch flexibility. Also, Euclidean distances with the Ward's method were used for cluster analysis, as a metric to measure the dissimilarity and similarity among the studied accessions, based on the phenotypic data (Figure 3). The dendrogram revealed two main clusters. The first cluster (I) contained seven accessions. The second cluster contained the rest accessions, forming two sub‐clusters. Also, according to the population analysis (Figure 4), the studied populations were placed into four groups. The Arisman, Shoor, Zardenjan, and Khoya populations were placed into the first group, while Sagzi, Heidarabad, and Gavkhooni populations formed the second group. Besides, Ardestan, Dehzire, and Badrood populations were placed into the third group, while fourth group consisted of Nikabad, Dastjerd, Varzaneh, and Harand populations. The obtained data revealed the morphological diversity within the studied populations. High dissimilarity levels among the studied accessions showed high variability in the germplasm. The reason for such a high dissimilarity can be explained by a low probability of gene flow among the studied accessions.

The S*. rosmarinus* is a xerophytic desert salt‐tolerant plant having genes responsible for its resistance to salt and drought stresses. It can serve as a very useful tool in the hands of plant breeders to produce agricultural crops resistant to these stresses. It accumulates copper and manganese at nontoxic levels, and has a high level of protein (7%) and 80% digestible organic matters (Koocheki & Mahalati, 1994). With these nutritional properties, it can be used as forage for livestock especially for camels in severe dry and saline desert conditions. Further therapeutic properties of this plant should be explored, for example, for the treatment of acnes.

The leaves of *S. rosmarinus* accumulate a large amount of soda compounds which can be used in several industries such as making soaps and detergents, pottery, ceramics, in sugar factories (sugar crystalinization), and copper bleaching. The potential of this species in environmental protection such as wind break and preventing soil erosion should not be overlooked (Kurkova et al., 2002).

## 4 CONCLUSION

This is the first report on the application of morphological characteristics in the evaluation of the phenotypic variation of *S. rosmarinus*. This study presented high phenotypic diversity of *S. rosmarinus* germplasm. The screening of the natural germplasm of wild *S. rosmarinus* is one of the most important perquisites to conserve and domesticate these valuable species. The presence of variation is crucially vital to preserving the evolutionary ability to live under a dynamic climatic condition. The phenotypic diversity among these individuals could provide useful information for conservation and selection of cross‐parents in breeding.

## CONFLICT OF INTEREST

The authors declare no conflict of interest.

## RESEARCH INVOLVING HUMAN PARTICIPANTS AND/OR ANIMALS

None.

## INFORMED CONSENT

None.

## Data Availability

The data that support the findings of this study are available from the corresponding author upon reasonable request.

## References

[fsn32964-bib-0001] Amiraslani, F. , & Dragovich, D. (2011). Combating desertification in Iran over the last 50 years: An overview of changing approaches. Journal of Environmental Management, 92, 1–13. 20855149 10.1016/j.jenvman.2010.08.012

[fsn32964-bib-0002] Badenes, M. L. , Martinez‐Calvo, J. , & Llacer, G. (2000). Analysis of a germplasm collection of loquat (*Eriobotrya japonica* Lindl.). Euphytica, 114, 187–194.

[fsn32964-bib-0003] Deymeh, H. , Shadizadeh, S. R. , & Motafakkerfard, R. (2012). Experimental investigation of *Seidlitzia rosmarinus* effect on oil–water interfacial tension: Usable for chemical enhanced oil recovery. Scientia Iranica, 19, 1661–1664.

[fsn32964-bib-0004] Hadi, M. R. (2009). Biotechnological potentials of *Seidlitzia rosmarinus*: A mini review. African Journal of Biotechnology, 8, 2429–2431.

[fsn32964-bib-0005] Hammer, Ø. , Harper, D. A. T. , & Ryan, P. D. (2001). PAST: Paleontological statistics software package for education and data analysis. Palaeontologia Electronica, 4(1), 9. http://palaeoelectronica.org/2001_1/past/issue1_01.htm

[fsn32964-bib-0006] Jafari, M. , ZareChahouki, M. A. , Tavili, A. , & Azarnivand, H. (2003). Soil vegetation relationships in Hoz‐e‐Soltan region of Qom Province, Iran. Pakistan Journal of Nutrition, 2, 329–334.

[fsn32964-bib-0007] Jongbloed, M. (2003). The comprehensive guide to the wild flowers of The United Arab Emirates. Environmental Research and Wildlife Development Agency.

[fsn32964-bib-0008] Koocheki, A. , & Mahalati, M. N. (1994). Feed value of some halophytic range plants of arid regions of Iran. Kluwer Academic Publishers.

[fsn32964-bib-0009] Kurkova, E. B. , Kalinkina, L. G. , Baburina, O. K. , Myasoedov, N. A. , & Naumova, T. G. (2002). Responses of *Seidlitzia rosmarinus* to salt stress. Biology Bulletin of the Russian Academy of Sciences, 29(3), 221–229. 12071052

[fsn32964-bib-0010] Mahmoodi, T. , Khoshhal, J. , Mousavi, S. H. , & Pourkhosravani, M. (2013). A comparative evaluation of adaptation of nebkas to stabilize sand dunes of the desert in Sirjan using AHP model. Iran Journal of Environmental Erosion Research, 2, 24–38.

[fsn32964-bib-0011] Norusis, M. J. (1998). SPSS/PC advanced statistics. SPSS Inc.

[fsn32964-bib-0012] Sagheb‐Talebi, K. , Sajedi, T. , & Pourhashemi, M. (2014). Forests of Iran: A treasure from the past, a hope for the future (Vol. 152). Springer, Berlin Heidelberg publisher.

[fsn32964-bib-0013] SAS® Procedures . (1990). Version 6 (3rd ed.). SAS Institute.

[fsn32964-bib-0014] Swingle, R. , Glenn, E. , & Squires, V. (1996). Growth performance of lambs fed mixed diets containing halophyte ingredients. Animal Feed Science and Technology, 63, 137–148.

[fsn32964-bib-0015] Yang, W. Z. , Jin, H. , Li, W. Y. , Zhang, Z. H. , Zhao, Z. L. , & Zhang, J. Y. (2013). A study on phenotypic diversity in different populations of endangered *Coptis teeta* wall of Yunnan. Journal of Yunnan University, 35, 719–726.

